# Orphan nuclear receptor *ftz-f1 (NR5A3)* promotes egg chamber survival in the *Drosophila* ovary

**DOI:** 10.1093/g3journal/jkab003

**Published:** 2021-01-23

**Authors:** Allison N Beachum, Kaitlin M Whitehead, Samantha I McDonald, Daniel N Phipps, Hanna E Berghout, Elizabeth T Ables

**Affiliations:** Department of Biology, East Carolina University, Greenville, NC 27858, USA

**Keywords:** oocyte, follicle cells, nuclear hormone receptor, oogenesis

## Abstract

Gamete production in mammals and insects is controlled by cell signaling pathways that facilitate communication between germ cells and somatic cells. Nuclear receptor signaling is a key mediator of many aspects of reproduction, including gametogenesis. For example, the NR5A subfamily of nuclear receptors is essential for gonad development and sex steroid production in mammals. Despite the original identification of the NR5A subfamily in the model insect *Drosophila melanogaster*, it has been unclear whether *Drosophila* NR5A receptors directly control oocyte production. *Ftz-f1* is expressed throughout the ovary, including in germline stem cells, germline cysts, and several populations of somatic cells. We show that *ftz-f1* is required in follicle cells prior to stage 10 to promote egg chamber survival at the mid-oogenesis checkpoint. Our data suggest that egg chamber death in the absence of *ftz-f1* is due, at least in part, to failure of follicle cells to exit the mitotic cell cycle or failure to accumulate oocyte-specific factors in the germline. Taken together, these results show that, as in mammals, the NR5A subfamily promotes maximal reproductive output in *Drosophila*. Our data underscore the importance of nuclear receptors in the control of reproduction and highlight the utility of *Drosophila* oogenesis as a key model for unraveling the complexity of nuclear receptor signaling in gametogenesis.

## Introduction

Oogenesis is a multistage process requiring precise spatiotemporal cellular communication. A variety of paracrine and endocrine signals enable a coordinated response of ovarian cells to intrinsic and physiological cues. In mice, humans, and insects, nutritionally-responsive growth factors and reproductive steroids control cell proliferation, survival, and the timing of oocyte development (Grive and Freiman 2015; Uryu *et al.* 2015; Ables and Drummond-Barbosa 2017; Wang *et al.* 2017; Chou and Chen 2018; Richards 2018; Swevers 2019; Yatsenko and Rajkovic 2019). Cells respond to reproductive steroids and other nutritionally regulated small molecules via the nuclear receptor superfamily of ligand-gated transcription factors (King-Jones and Thummel 2005; Pardee *et al.* 2011). Due to their unique ability to bind DNA in response to circulating cues, nuclear receptors are key transcriptional regulators of gene expression in diverse species (Evans and Mangelsdorf 2014).

The mammalian NR5A subgroup of nuclear receptors has been implicated in a variety of reproductive contexts, including sex determination, gonad development, and ovulation (Yazawa *et al.* 2015; Meinsohn *et al.* 2019). NR5A receptors are considered “orphan” nuclear receptors, able to bind phospholipids but also adopt an active conformation in the absence of a ligand (Krylova *et al.* 2005; Yoo et al. 2011; Lu *et al.* 2013; Musille *et al.* 2013; Daffern *et al.* 2018). Mammalian NR5A members Liver Receptor Homolog 1 (LRH-1) and Steroidogenic Factor-1 (SF-1) bind the same DNA sequence motif but regulate distinct sets of target genes in multiple tissues associated with the reproductive axis (Meinsohn *et al.* 2019). In the mouse ovary, LRH-1 is critical for granulosa cell proliferation, ovulation, and proper formation and function of the corpus luteum (Duggavathi *et al.* 2008; Bertolin *et al.* 2014; Bertolin *et al*. 2017; Meinsohn *et al.* 2018). Global knockout of *SF-1* abrogated gonad and adrenal development, resulting in early perinatal lethality (Parker *et al.* 1996), whereas ovarian granulosa cell-specific deletion of *SF-1* resulted in sterility, fewer oocytes, and decreased follicle growth (Pelusi *et al.* 2008; Buaas *et al.* 2012). Uterine morphology and endometrial establishment were also compromised in *SF-1* and *LRH-1* knockout models, resulting in infertility or unsuccessful embryo implantation (Pelusi *et al*. 2008; Zhang et al. 2013).

In contrast to the 48 nuclear receptors in most mammals, the *Drosophila melanogaster* genome encodes only 18 nuclear receptor genes, representing six subfamilies of receptors with minimal genetic redundancy (King-Jones and Thummel 2005). Although some nuclear receptors, such as the steroid-responsive Ecdysone Receptor (EcR) and nitric oxide-responsive Ecdysone-induced protein 75B (E75), are essential for female reproduction, it is not fully understood whether or how other receptors mediate oogenesis (Ables and Drummond-Barbosa 2017). The *Drosophila* genome encodes two conserved NR5A family members, *Hormone receptor-like in 39* (*Hr39*) and *ftz transcription factor 1* (*ftz-f1*) (King-Jones and Thummel 2005). *Hr39* is necessary for reproductive tract development, but is not intrinsically required in the ovarian epithelium for oogenesis (Allen and Spradling 2007; Sun and Spradling 2012; 2013; Ables *et al.* 2016). Hr39 and Ftz-f1 contain DNA binding domains that are structurally conserved with mammalian homologs and bind similar DNA sequences as SF-1 and LRH-1 (King-Jones and Thummel 2005). Interestingly, *SF-1* and *LRH-1* can functionally replace *ftz-f1* in transcriptional activation of embryonic genes, but only *LRH-1* can rescue loss of *HR39* in reproductive tissues (Lu *et al.* 2013). Crystal structure analysis suggests that Ftz-f1 can bind phospholipids, but also can be activated in the absence of ligand binding (Yoo *et al.* 2011; Daffern *et al.* 2018).

*Ftz-f1* is well-known in insects for its role in embryonic patterning, metamorphosis, and pupal development. In *Drosophila*, the *ftz-f1* gene locus encodes two isoforms. α-*ftz-f1* is maternally loaded in the egg and functions as an essential co-factor with *fushi tarazu* for proper embryo anterior/posterior patterning (Ueda *et al.* 1992; Guichet *et al.* 1997; Schwartz *et al.* 2001). β-*ftz-f1* is necessary at metamorphosis for remodeling of larval tissues and for ecdysone-induced gene expression (Broadus *et al.* 1999; Yamada *et al.* 2000; Boulanger *et al.* 2011). β-*ftz-f1* is necessary for cholesterol uptake and conversion to ecdysone in the larval prothoracic gland, suggesting functional conservation with SF-1 in steroid hormone biosynthesis (Parvy *et al.* 2005; Talamillo *et al.* 2013). Intriguingly, *ftz-f1* is also essential for oogenesis in the mosquito *Aedes aegypti* and the red flour beetle *Tribolium castaneum* (Li *et al.* 2000; Xu *et al.* 2010). More recently, *ftz-f1* was shown to be required in follicle cells during the final stages of oogenesis to promote ovulation (Knapp *et al.* 2020).

**Figure 1:**
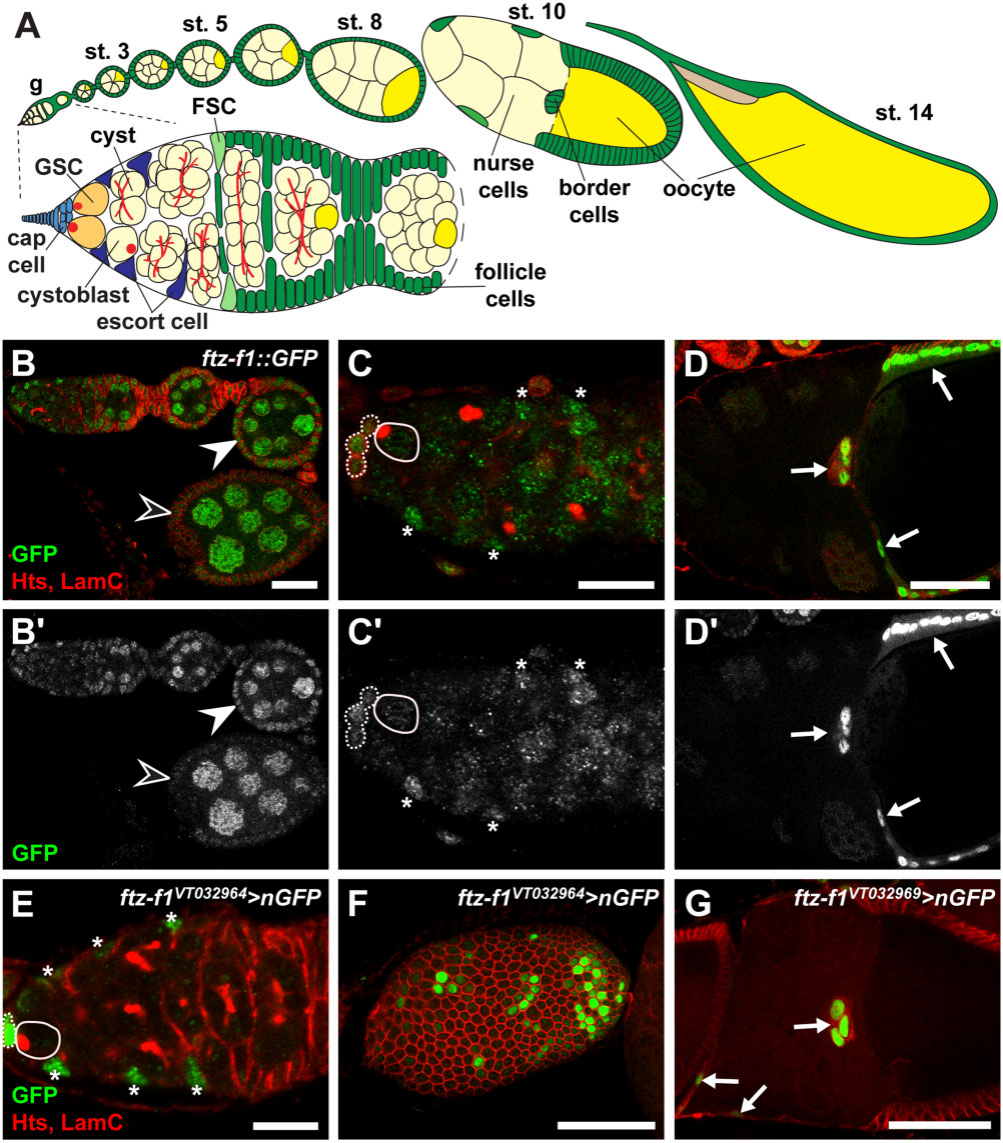
*ftz-f1* is expressed in the ovarian epithelium. (A) The *Drosophila* ovary is made of 14-16 ovarioles (top), each consisting of a germarium and progressively older egg chambers. Germline stem cells (GSCs) are housed in the germarium (enlarged below) and divide asymmetrically away from cap cells (light blue). GSC daughters, called cystoblasts, divide four additional times to form 16-cell cysts composed of 15 nurse cells and one oocyte (yellow). Escort cells (dark blue) navigate the cysts towards the follicle stem cells (FSC, light green) and follicle cells (kelly green). Cysts are encapsulated by follicle cells to form egg chambers that pinch off from the germarium and progress through 14 stages of oogenesis. (B-D') Single plane image of ovariole (B, B'), germarium (C, C'), and stage 10B egg chamber (D, D') from *ftz-f1::GFP* females labeled with anti-GFP (green; Ftz-f1::GFP), anti-Hts (red; fusomes and follicle cell membranes), and anti-LamC (red; nuclear envelopes). GSCs are outlined in solid white lines; cap cells are outlined in dotted yellow lines. Asterisks indicate GFP-positive escort cells. B'-D’ depict the GFP channel of the image above. Filled arrowhead denotes GFP-positive follicle cells; open arrowhead denotes GFP-negative follicle cells. Arrows indicate GFP-positive border cells and main body follicle cells. (E–G) Gal4 lines *ftz-f1^VT032964^* (E, F) and *ftz-f1^VT032969^* are sufficient to drive reporter expression (green) in escort cells (asterisks in E), main body follicle cells (F) and stage 10A border cells (arrows in G). Scale bars, 10 μm (C, C', E), 20 μm (B, B'), or 50 μm (D, D', F, G).

Given the importance of NR5A family members in oogenesis in other species, it is somewhat surprising that Ftz-f1 has not been well-studied in *Drosophila*. Each female fly contains two ovaries, made of 14-16 ovarioles which are strings of progressively mature egg chambers or follicles (McLaughlin and Bratu 2015; Hinnant *et al.* 2020). Each egg chamber contains a cyst of 16 interconnected germ cells surrounded by somatic follicle cells. Egg chamber development begins in the germarium, located at the anterior of each ovariole (Figure 1A). Here, germline stem cells (GSCs) divide asymmetrically to produce one self-renewing GSC daughter and another daughter cell (called a cystoblast) capable of differentiation. Cystoblasts divide four times with incomplete cytokinesis to generate 2-,4-,8-, and 16-cell cysts. As germ cells prepare for final rounds of mitosis, three to four cyst cells begin to build synaptonemal complexes necessary for meiosis; concurrent expression of oocyte-specific proteins begins in 8- and 16- cell cysts (Hughes *et al.* 2018; Hinnant *et al.* 2020). Soon after the completion of the last mitotic division, only one cyst cell remains in meiosis and maintains expression of oocyte-specific factors. The remaining 15 cells adopt a nurse cell fate, which transcribe maternal mRNAs and transport them into the oocyte for later use in early embryogenesis.

As in mammals, *Drosophila* oocytes are intimately associated with somatic cells that ensure proper oocyte differentiation and survival. Mitotically dividing germ cells are guided through the germaria by somatic escort cells (Banisch *et al.* 2017). Cessation of germ cell mitosis coincides with a transfer of 16-cell cysts from escort cells to pre-follicle cells, which originate from a second population of stem cells, the follicle stem cells (Rust and Nystul 2020). Pre-follicle cells migrate around cysts and interdigitate to separate newly forming egg chambers (Lovegrove *et al.* 2019). Pre-follicle cell proliferation creates more separation between the germarium and the developing egg chambers, eventually budding off to form individual egg chambers. As they leave the germarium, pre-follicle cells differentiate into the main body, polar, and stalk cells (Duhart *et al.* 2017). Main body follicle cells make up most of the egg chamber and are proliferative during stages 1–6 to cover the growing cyst in an epithelial monolayer (Jia *et al.* 2015). At stage 6, concomitant with the onset of yolk uptake into the oocyte, the follicle cells exit mitosis and shift to endocycling, wherein repeated rounds of DNA synthesis occur without mitosis. From stages 10B to 13, follicle cells cease whole-genome duplication and instead amplify selective genomic regions essential for eggshell formation. At stage 14, the egg chamber is characterized as a mature egg and is ready to be ovulated.

In this study, we show that *ftz-f1* is essential during the early stages of egg chamber growth for optimal *Drosophila* female fecundity, underscoring the evolutionarily conserved role of the NR5A family in female reproduction. Using cell-type-specific loss-of-function techniques, we find that *ftz-f1* promotes egg chamber survival. In follicle cells, *ftz-f1* promotes the integrity of the follicle cell monolayer, non-autonomously preventing caspase-mediated cell death of the underlying germ cells. Moreover, when *ftz-f1* is depleted simultaneously from the follicle cells and the germline, egg chambers fail to progress past stage 4 due to germ cell death. We provide evidence that *ftz-f1* may promote egg chamber survival, at least in part, by two distinct mechanisms. In follicle cells, *ftz-f1* promotes mitotic exit at stage 6. In germ cells, *ftz-f1* controls accumulation of oocyte-specific factors, likely impacting oocyte polarity. We postulate that the combined roles of *ftz-f1* in the developing germline and soma function interdependently to sustain egg chamber growth and survival. With these data, our study adds to a growing body of literature emphasizing the multifaceted roles of nuclear receptors in the control of female reproduction.

## Materials and methods

### *Drosophila* husbandry and culture

All *Drosophila* stocks were maintained on standard cornmeal/molasses/yeast medium (Genesee Scientific, Nutri-Fly-MF) at 22°C–25°C. Genes/alleles with multiple names are referenced using FlyBase nomenclature (www.flybase.org; last accessed October 2020) for simplicity. Except where noted, female flies were collected one to two days after eclosion and maintained on standard medium supplemented with wet yeast paste for 2–3 days (changed daily) at 25°C prior to ovary dissection. For assessment of Ftz-f1 expression in the ovary, we used transgenic line *ftz-f1^sfGFP, Tag: FLAG^* (*ftz-f1::GFP*), which carries a bacterial artificial chromosome containing the entire *ftz-f1* gene locus and surrounding regulatory DNA, including an sfGFP-Tag: FLAG cassette introduced at the C-terminal end of the *ftz-f1* coding region (Bloomington stock #38645; R. Spokony). Expression of *ftz-f1* in escort cells and follicle cells was further confirmed using *P{VT032964-GAL4}attP2* (*VT032964-Gal4*) and *P{VT032969-GAL4}attP2* (*VT032969-Gal4*), in which ∼2 kB of intronic sequence from *ftz-f1-RB* is fused upstream of a *Drosophila* synthetic core promoter and *GAL4* (Kvon *et al.* 2014; McDonald *et al.* 2019). *VT032964-Gal4* and *VT032969-Gal4* were crossed with *P{w+mC=UAS-lacZ.NZ}J312* (*UASt-lacZ*; Bloomington stock #3956) to confirm driver expression. Balancers and other genetic tools are described in FlyBase (Thurmond *et al.* 2019).

### Tissue-specific RNA interference

For knock-down of *ftz-f1* in somatic cells, we used the following RNA interference (RNAi) lines: *y^1^ v^1^; P{TRiP.JF02738}attP2* (*ftz-f1^JF^*; Bloomington stock #27659) (Li *et al.* 2014), *P{KK108995}VIE-260B* (*ftz-f1^KK^*; Vienna stock #104463), and *P{TRiP.HMS00019}attP2* (*ftz-f1^HMS^*; Bloomington stock #33625*)*. To limit *Gal4* expression specifically to adult follicle cells (thus circumventing developmental lethality), we used the *Gal4/Gal80^ts^* system (McGuire *et al.* 2003). Flies bearing *tj-Gal4; tubGal80^ts^* (*tj-Gal4;*  Sahai-Hernandez and Nystul 2013) (kindly provided by E. Matunis) were raised at 18°C and then shifted to 29°C at eclosion to induce expression of the *UAS-RNAi* constructs as described (Blake *et al.* 2017). Driver expression was confirmed using y^1^ w*; P{w^+mC^=UAS-mCD8::GFP.L}LL5 (*UAS-mCD8::GFP*; Bloomington stock #5137). Egg chambers were staged based on size, shape, yolk accumulation, and germ cell nuclear morphology as described (King 1970; Spradling 1993).

### Egg production and viability assays

Egg-laying assays were conducted to assess female fertility. Five young (∼24 h old) females were mated with five age-matched wild-type males in bottles with Nutri-fly Grape Agar Premix (Genesee Scientific) plates topped with a small amount of wet yeast paste and maintained at 25°C. Bottles were set in triplicate for each control and experimental genotype. Agar plates were replaced every 24 h for ten days. The number of eggs were counted for each plate and divided by the number of females in the bottle. During the first two days of the assay, eggs were removed from their original plate to a fresh grape agar plate and allowed to develop at 25°C for an additional 24 h. Hatch rates were calculated by dividing the number of eggs that completed hatching by the total number of eggs in the assay.

### Genetic mosaic generation

For genetic mosaic analyses using *flippase* (*FLP*)/*FLP recognition target* (*FRT*) (Xu and Rubin 1993), we obtained mutant alleles *ftz-f1^ex7^* and *ftz-f1^19^* on *FRT79D*-containing chromosome arms (kindly provided by L. Pick, C. Woodard, and J. Dura). Genetic mosaics were generated by *FLP/FRT*-mediated recombination in 2- to 3- d old females carrying a *ftz-f1* mutant allele in trans to a wild-type allele (linked to a *nuclear-GFP* marker; kindly provided by M. Buszczak) on homologous *FRT* arms, and a *hs-FLP* transgene, as described (Laws and Drummond-Barbosa 2015). Flies were heat shocked at 37°C two times per day for 3 days, and incubated at 25°C for 8 or 12 days with transfers to freshly yeasted vials occurring every other day (standard media supplemented with dry yeast, and wet yeast paste on the last 3 days prior to dissection). Wild-type alleles were used for generation of control mosaics. Germline cysts in the germarium were identified based on fusome morphology (de Cuevas and Spradling 1998; Ong and Tan 2010) and egg chambers were staged based on size and nuclear morphology as described (King 1970; Spradling 1993). Additional phenotypes, including egg chamber death, were noted in comparison with adjacent GFP-positive wild-type cells in stage-matched or adjacent egg chambers.

### Immunostaining and microscopy

Ovaries were prepared for immunofluorescence microscopy as described (Ables and Drummond-Barbosa 2013). Ovaries were dissected and ovarioles teased apart in Grace's medium without additives (Caisson Labs) and fixed in 5.3% formaldehyde (Ted Pella Inc, 18505) in Grace's medium for 13 min at room temperature. They were then washed extensively in phosphate-buffered saline (PBS, pH 7.4; Fisher) with 0.1% Triton X-100, and blocked for 3 h in blocking solution [5% bovine serum albumin (Sigma), 5% normal goat serum (MP Biomedicals), and 0.1% Triton X-100 in PBS] at room temperature. To detect cells in S phase, dissected ovaries were kept intact (no teasing of ovarioles) and incubated for 1 h at room temperature in Grace's media containing 10 μM 5-ethynyl-2′-deoxyuridine (EdU; Life Technologies). Ovaries labeled with EdU were then fixed, ovarioles teased apart, washed extensively in 0.1% Triton X-100 in PBS, and blocked as described above. The following primary antibodies were used overnight at 4°C: mouse anti-Hts [1B1, Developmental Studies Hybridoma Bank (DSHB); 1:10], mouse anti-Lamin C (LamC) (LC28.26, DSHB; 1:100), chicken anti-GFP (ab13970, Abcam; 1:2000), mouse anti-Orb (4H8/6H4, DSHB; 1:100), mouse anti-BicD (1B11/4C2, DSHB; 1:10), rabbit anti-phosphoHistone H3 (06-570, Millipore; 1:200), rabbit anti-Dcp1 (37729, Cell Signaling; 1:100), and chicken anti-β-Galactosidase (ab9361, Abcam; 1:2000). Following an overnight incubation at 4°C with Alexa Fluor 488-, 568-, or 633- conjugated goat species-specific secondary antibodies (Life Technologies; 1:200), EdU was detected (if necessary) using AlexaFluor-594 via Click-It chemistry, following the manufacturer's recommendations (Life Technologies). Ovaries were counter-stained with 0.5 mg/ml 49-6-diamidino-2-phenylindole (DAPI) (Sigma) to identify nuclei or phalloidin-AlexaFluor-647 (Life Technologies; 1:400) to visualize F-actin. Ovaries were mounted in 90% glycerol containing 20 mg/ml n-propyl gallate (Sigma). Confocal Z-stacks (1 μm optical sections) were collected with a Zeiss LSM700 microscope using Zeiss ZEN software. Images were analyzed, and minimally and equally enhanced via histogram using Zeiss ZEN software.

### Statistical analysis

All experiments were performed in triplicate from independent genetic crosses, using at least 10 ovaries per replicate. Statistical analysis was performed in Prism (GraphPad, Inc.) and Excel (Microsoft) software. Statistical differences between one control group and one experimental group were analyzed by Student’s two-tailed t-test or Chi-square analyses (*P <* *0.05, **P <* *0.01, ***P <* *0.001). Bar graphs show averages plus/minus the standard error of the mean (SEM). Sample values (*n*) are presented on graphs in or above bars and represent the number of cells, ovarioles, or germaria examined as indicated.

### Data availability

Fly strains and plasmids are available upon request. The authors affirm that all data necessary for confirming the conclusions of the article are present within the article and figures.

## Results

### *ftz-f1* is widely expressed in the adult ovary

The nuclear receptor encoded by *ftz-f1* is expressed in a variety of tissue types and required for multiple developmental processes, including adult head eversion, leg elongation, salivary gland cell death, and ovulation (Broadus *et al.* 1999; Knapp *et al.* 2020). To assess *ftz-f1* expression in the ovarian epithelium, we took advantage of a reporter transgene in which a bacterial artificial chromosome corresponding to the entire *ftz-f1* gene locus (and surrounding DNA) was engineered to include *green fluorescent protein* (*GFP*) inserted in frame on the C-terminal end of the *ftz-f1* locus (*ftz-f1::GFP*; Figure 1, B–D’; R. Spokony, personal communication). The fusion protein created by *ftz-f1::GFP* is likely functional, as it is sufficient to partially rescue hypomorphic (*ftz-f1^19^*) and null (*ftz-f1^ex7^*) mutants to adulthood. To identify cell type-specific expression of Ftz-f1::GFP in the adult ovary, we performed co-immunofluorescence with antibodies against the adducin-like protein Hu li tai shao (Hts), which is abundant in germ cell fusomes and in the cytoskeleton of follicle cells (Zaccai and Lipshitz 1996; de Cuevas and Spradling 1998), and LaminC (LamC), to visualize nuclear envelopes (particularly useful for identifying cap cells). We found that Ftz-f1::GFP is broadly expressed at varying levels throughout the adult ovary, including the germarium and follicles at each stage of oogenesis (Figure 1, B and B’), and predominantly localized to nuclei. In the germarium, Ftz-f1::GFP was detectable in GSCs, cystoblasts, and dividing cysts, but was less abundant than in the surrounding somatic cap cells and escort cells (Figure 1, C and C’). We also observed Ftz-f1::GFP in pre-follicle cells and differentiated follicle cells in egg chambers from stage 2 to 5 (Figure 1, B and B’). Intriguingly, Ftz-f1::GFP was largely absent from follicle cells in egg chambers from stages 6–8 (open arrowhead in Figure 1, B and B’), but was abundant in post-migratory follicle cells in stage 10 (arrows in Figure 1, D and D’). This pattern is consistent with a recent study in which high levels of Ftz-f1 protein were detected transiently in follicle cells in stages 10–12 (Knapp *et al.* 2020). To further validate the *ftz-f1* expression in early follicle cells, we used two *ftz-f1* enhancer trap lines (*ftz-f1^VT032964^* and *ftz-f1^VT032969^*) that are sufficient to drive reporter gene expression in the ovary (McDonald *et al.* 2019). Both reporters correspond to α-*ftz-f1* intronic DNA, and *ftz-f1^VT032964^* overlaps a previously characterized β-*ftz-f1* enhancer element (Kageyama *et al.* 2003; McDonald *et al.* 2019). Similar to Ftz-f1::GFP expression, *ftz-f1* enhancer trap lines were able to drive expression of a *lacZ* reporter in cap cells and escort cells in the germarium (*ftz-f1^VT032964^*; Figure 1E) and some main body follicle cells in stages 5–10B (both reporters; Figure 1, F and G). Interestingly, neither enhancer trap line was sufficient to completely recapitulate the Ftz-f1::GFP follicle cell expression pattern. We speculate that multiple enhancer elements are required to fully activate expression. Our results show that *ftz-f1* is expressed in germ cells and somatic cells prior to vitellogenesis, albeit at lower levels than in stages 10–12 follicle cells.

### *ftz-f1* is essential in the soma for female fertility and early embryo viability

**Figure 2:**
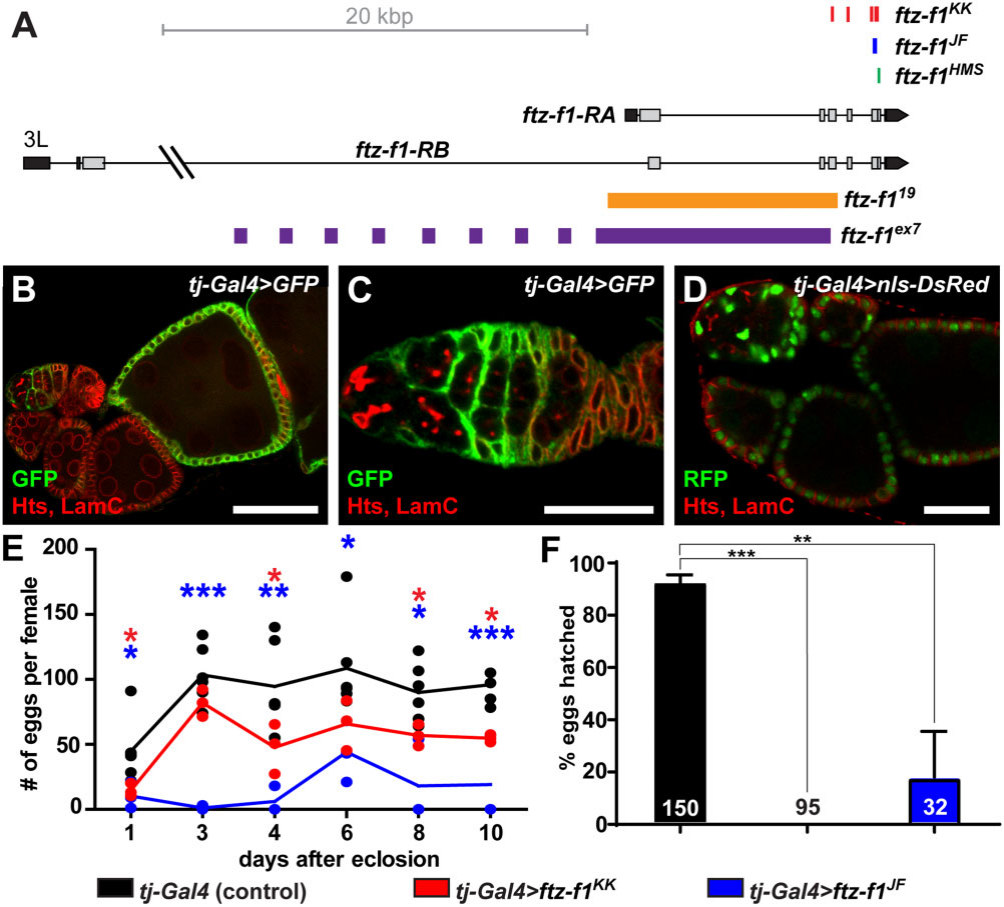
*ftz-f1* is necessary in ovarian follicle cells for optimal female fecundity. (A) Schematic of the *ftz-f1* locus with mutant alleles and RNAi constructs used in this study. Orange (*ftz-f1^19^*) or purple (*ftz-f1^ex7^*) lines represent genomic regions deleted in indicated mutant alleles; for RNAi lines, colored dashes (red, *ftz-f1^KK^*; blue, *ftz-f1^JF^*; green, *ftz-f1^HMS^*) indicate gene regions targeted by the corresponding shRNA. (See Materials and Methods for full RNAi description.) (B–D) *tj-Gal4* drives expression in ovarian somatic cells. Single z-plane images of ovarioles (B, D) or a germarium (C) from *tj-Gal4>UASt-GFP* (B, C) or *tj-Gal4>UASt-nls-DsRed* (D) females. Ovarioles were immunostained for anti-GFP (green, B, C) or anti-DsRed (green, D), anti-Hts (red; fusomes and follicle cell membranes), and anti-LamC (red; nuclear envelopes). Scale bars, 20 μm (C-D) or 50 μm (B). (E) Number of eggs laid per day by age-matched control (*tj-Gal4* alone; black) or *ftz-f1* knock-down (*tj-Gal4>UAS-ftz-f1^KK^*, red; *tj-Gal4>UAS-ftz-f1^JF^*, blue) females. Each dot represents an average number of eggs laid by five females (n = 6 independent experiments for driver control; n = 3 independent experiments each for *tj-Gal4>UAS-ftz-f1^KK^* and *tj-Gal4>UAS-ftz-f1^JF^*). Lines represent the average number of eggs laid by each genotype. (F) Eggs laid by control and *ftz-f1* knock-down females (fertilized by wild-type males) were monitored for hatching. Numbers in bars represent the total number of eggs analyzed. *P < 0.05, **P < 0.01, ***P < 0.001; Student’s two-tailed *t*-test.

Previous studies showed that Ftz-f1 is maternally deposited into oocytes and functions as a co-factor with *fushi tarazu* to establish proper embryonic patterning (Guichet *et al.* 1997; Yu *et al.* 1997; Hou *et al.* 2009). Transient expression of Ftz-f1 in stage 10-12 follicle cells is also necessary for the final stages of follicle cell maturation and oocyte ovulation (Knapp *et al.* 2020). As we observed Ftz-f1::GFP expression in ovarian epithelial cells prior to stage 10, we hypothesized that *ftz-f1* could impact oocyte development via an essential role earlier in oogenesis. Flies harboring homozygous mutant alleles of *ftz-f1* do not survive to adulthood (Lavorgna *et al.* 1993; Guichet *et al.* 1997; Yu *et al.* 1997; Broadus *et al.* 1999; Yamada *et al.* 2000). We therefore used short hairpin interfering RNA (RNAi) to specifically reduce *ftz-f1* function in ovarian cells via the tissue-specific *UAS-Gal4* system (Figure 2A) (Ni *et al.* 2011). Somatic driver *tj-Gal4* is strongly expressed in somatic cells in the germarium as well as main body follicle cells in stages 6–10 (Figure 2, B and C), and weakly in follicle cells in stages 1–5 (Figure 2D). Because *tj-Gal4* is also expressed in the developing nervous system prior to adulthood, we combined the *Gal4* system with the temperature-sensitive *Gal80^ts^* to suppress Gal4 activity until after eclosion to avoid developmental lethality. To determine whether *ftz-f1* is necessary in ovarian somatic cells to support oogenesis, we quantified egg deposition by *ftz-f1* mutant females mated to wild-type males as a physiological assessment of oocyte production (Figure 2E). Females in which *ftz-f1* was knocked-down in ovarian somatic cells prior to stage 10 using *tj-Gal4* laid fewer eggs compared to driver-alone or RNAi-alone controls (Figure 2E). We then asked whether knock-down of *ftz-f1* in ovarian somatic cells could support embryonic development post-fertilization. Eggs were collected 24 h after mating, allowed to develop at 25°C for an additional 24 h, and monitored for deflation, a sign of embryo hatching. Interestingly, oocytes produced by *tj-Gal4>ftz-f1^RNAi^* females did not support embryonic development as well as wild-type controls (Figure 2F). Taken together, these results show that *ftz-f1* is necessary in ovarian somatic cells prior to their final maturation for proper female fecundity.

### *ftz-f1* is necessary in follicle cells for egg chamber survival

**Figure 3:**
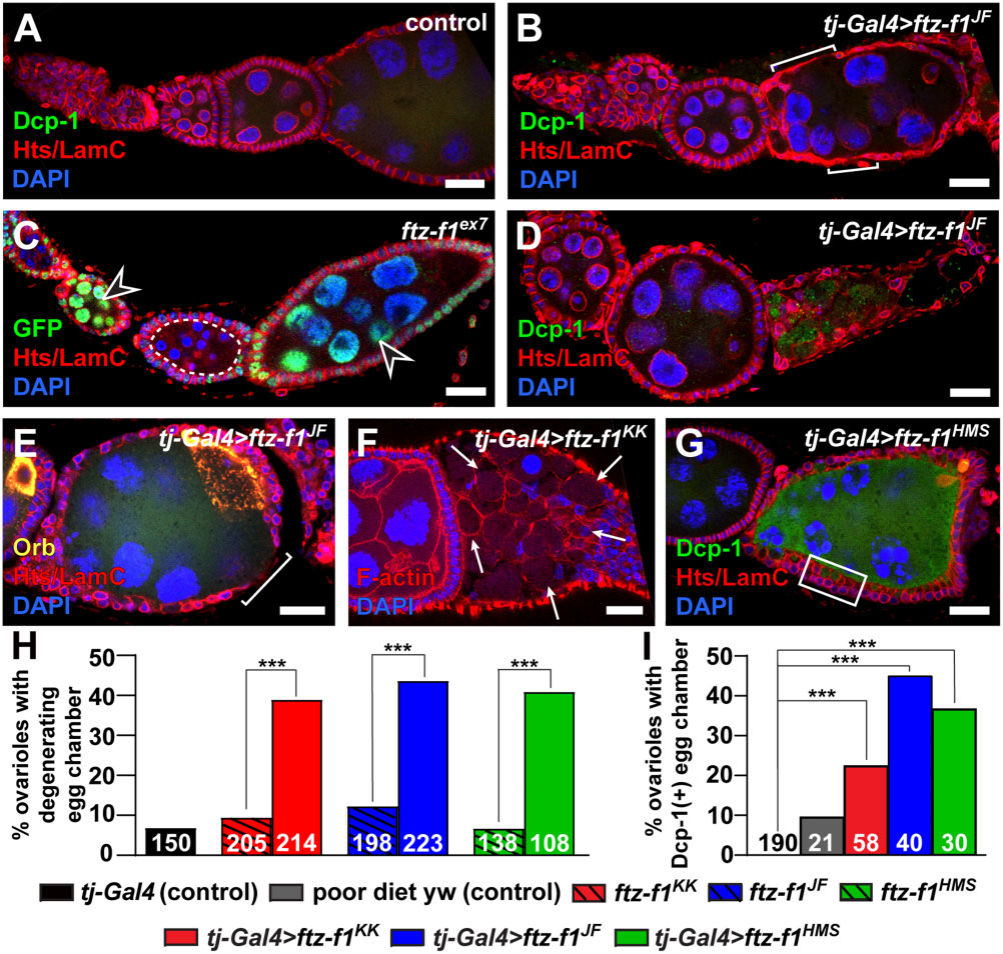
*ftz-f1* promotes egg chamber survival. (A, B, D, G) Single z-plane images of driver control (A) or *tj-Gal4>ftz-f1^RNAi^* ovarioles (B, D, G) immunostained with anti-Dcp-1 (green; activated caspase), anti-Hts (red; fusomes and follicle cell membranes) and anti-LamC (red; nuclear envelopes), and DAPI (blue, nuclei). (C) Mosaic *ftz-f1^ex7^ FRT79D* ovariole labeled with anti-GFP (green, wild-type cells), anti-Hts and anti-LamC (red), and DAPI (blue). Homozygous *ftz-f1^ex7^* mutant cysts are outlined in dotted white lines; open arrowheads denote GFP-positive (wild-type) cysts. (E) Stage 7 egg chamber from a *tj-Gal4>ftz-f1^JF^* ovariole labeled with anti-Orb (yellow, oocytes), anti-Hts and anti-LamC (red), and DAPI (blue). (F) Degenerating egg chamber from a *tj-Gal4>ftz-f1^KK^* ovariole labeled with phalloidin (red, F-actin) and DAPI (blue). Scale bars, 20 μm. (H, I) Percent of ovarioles containing an abnormal or degenerating egg chamber (H) or Dcp-1-positive egg chamber (I). Numbers in bars represent the total number of ovarioles analyzed. ***P < 0.001; chi-squared test.

Somatic follicle cells enwrap germline cysts as they exit the germarium, forming an epithelial layer that surrounds each cyst and aids in oocyte growth, maturation, and vitellogenesis (McLaughlin and Bratu 2015; Duhart *et al.* 2017). Because knock-down of *ftz-f1* in somatic cells prior to stage 10 resulted in decreased egg production, we hypothesized that *ftz-f1* is needed in follicle cells to promote egg chamber growth or development. Using immunostaining for the follicle cell cytoskeletal protein Hts and DAPI to visualize nuclei, we assessed egg chamber development in ovarioles dissected from females five days after eclosion. Wild-type ovarioles consisted of four to five successively larger pre-vitellogenic egg chambers outside of the germarium and at least one vitellogenic (stage 10-14) egg chamber (Figure 3A). In pre-vitellogenic egg chambers, follicle cells formed an epithelial monolayer around the periphery of each egg chamber, and germline nurse cells in the center of each egg chamber had large nuclei with dense DNA (Figure 3A; see Figure 1A for schematic). In contrast, *ftz-f1* RNAi knockdown in follicle cells using three independent RNAi transgenes resulted in ovarioles with abnormally developed or degenerating pre-vitellogenic egg chambers (Figure 3, B and D–G; quantified in Figure 3H). Some egg chamber defects were relatively mild, including small gaps or thinning of the follicle cell monolayer (brackets in Figure 3, B and E) and did not appear to alter nurse cell nuclear morphology. In other egg chambers, the follicle cell monolayer was disorganized and collapsed (Figure 3D), follicle cells were stretched or rounded (as visualized by phalloidin staining for F-actin; arrows in Figure 3F), or follicle cell nuclei had been extruded out of the monolayer (box in Figure 3G). These more severe defects were typically accompanied by pyknotic nuclei in the underlying nurse cells (Figure 3, D, F, and G). Although it was not possible to confidently stage abnormal or degenerating egg chambers according to size, shape, or nurse cell nuclear morphology, we estimate that egg chamber defects in *tjGal4>ftz-f1^RNAi^* females occurred between stages 5–8 (based on the stage of the preceeding egg chamber).

As an independent assessment of *ftz-f1* function in somatic cells, we used genetic mosaic analysis using the *flippase/flippase recognition target* (*Flp/FRT*) system and two previously characterized loss-of-function *ftz-f1* alleles to generate *ftz-f1* mutant ovarian cells. Although *ftz-f1^ex7^* and *ftz-f1^19^* both harbor deletions in the gene locus, *ftz-f1^ex7^* lacks the exon encoding the Ftz-f1 DNA binding domain (Yamada *et al.* 2000; Suzuki *et al.* 2001; Fortier *et al.* 2003), suggesting that the mutation abrogates function of both *ftz-f1* isoforms (Figure 2A). Following clone induction, we compared *ftz-f1* mutant cells (identified by the absence of GFP) to adjacent wild-type cells (identified by a nuclear-localized GFP linked to the wild-type allele) (Figure 3C). Intriguingly, from more than 100 mosaic egg chambers, we found no egg chambers older than stage 6 (for *ftz-f1^ex7^* mosaics) or stage 7 (for *ftz-f1^19^* mosaics) containing a somatic cell layer composed solely of *ftz-f1* mutant cells. We did, however, find examples of egg chambers with both *ftz-f1* mutant somatic cells and mutant germ cells; these cases were all stage 6 or smaller and frequently had pyknotic germ cell nuclei, indicative of egg chamber death (Figure 3C). Taken together with our RNAi analyses, these results suggest that *ftz-f1* is necessary in follicle cells for the survival of mid-stage egg chambers.

Mid-oogenesis is sensitive to female starvation and serves as a checkpoint for caspase-mediated cell death and clearance of unfit egg chambers (Peterson *et al.* 2015). We therefore asked whether knock-down of *ftz-f1* in somatic cells resulted in caspase-dependent programmed cell death using antisera against cleaved Death caspase-1 (Dcp-1), an effector caspase (Laundrie *et al.* 2003; Peterson *et al.* 2015). As expected, we did not detect Dcp-1-positive ovarian cells when driver control female flies were fed a yeast-rich diet (Figure 3, A and I); however, about 10% of ovarioles contained a Dcp-1-positive egg chamber when control females were fed sugar only for two days (Figure 3I). In contrast, the percentage of ovarioles with a caspase-positive egg chamber after induction of *ftz-f1* knockdown in follicle cells was significantly greater than that of the controls (Figure 3, D, G, and I). Based on this result, we conclude that most egg chamber death in *tjGal4>ftz-f1^RNAi^* ovarioles can be attributed to inappropriate activation of cleaved caspases. Intriguingly, Dcp-1 immunoreactivity in *tjGal4>ftz-f1^RNAi^* egg chambers was not detected in follicle cells (Figure 3, B, C, and F). Rather, knock-down of *ftz-f1* in mutant follicle cells indirectly caused caspase-mediated cell death in the underlying germline (Figure 3G). These results suggest that *ftz-f1* is essential in follicle cells to protect germ cells from caspase-mediated cell death.

### *ftz-f1* promotes follicle cell mitotic exit at mid-oogenesis

**Figure 4:**
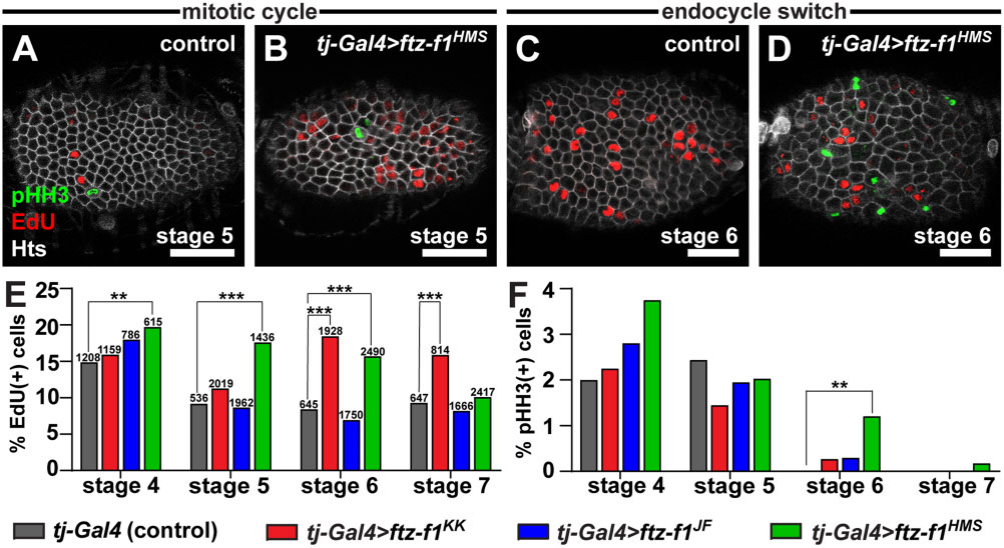
*ftz-f1* promotes timely mitotic exit in follicle cells. **(**A–D) Single z-plane images of control and *tj-Gal4>ftz-f1^HMS^* egg chambers at stage 5 or stage 6, labeled with anti-phospho-histone H3 (pHH3; green), EdU (red), and anti-Hts (white; follicle cell membranes). Scale bars, 20 μm. (E, F) Percent of follicle cells labeled with EdU (E) or pHH3 (F) in control and *tj-Gal4>ftz-f1^RNAi^* egg chambers. Numbers above bars in E represent the total number of follicle cells analyzed in both E and F. **P < 0.01, ***P < 0.001; chi-squared test.

During their development, main body follicle cells progress through three distinct modes of cell cycles (Jia *et al.* 2015). From the germarium to stage 5, follicle cells undergo a typical mitotic cell cycle, including G1, S, G2, and M phases marked by oscillation of mitotic cyclins, the S-phase indicator 5-ethynyl-2′-deoxyuridine (EdU), and the mitotic indicator phospho-Histone H3 (pHH3) (Figure 4A). At stage 6, main body follicle cells switch to endocycling, a specialized cell cycle in which cells alternate G and S phases for repeated rounds of DNA synthesis without cell division. This switch is marked by an abrupt disappearance of the mitotic cyclins and pHH3 (Figure 4, C and F). The final cell cycle switch occurs at stage 10B when the follicle cells exit endocycling, instead amplifying specific genomic regions necessary for proper eggshell morphogenesis (Deng *et al.* 2001; Klusza and Deng 2011; Jia *et al.* 2015).

Knock-down of *ftz-f1* in *tjGal4>ftz-f1^RNAi^* females resulted in egg chamber death predominantly during stages 5–8 (Figure 3). Given the correlation with the timing of the mitotic-to-endocycle switch, we hypothesized that egg chamber death might be a result of aberrant follicle cell proliferation. To investigate cell cycle control in wild-type and *tjGal4>ftz-f1^RNAi^* follicle cells, we co-labeled antisera against pHH3 with a fluorescently labeled EdU incorporated in dividing cells over a 1-h pulse (Figure 4). As expected, the percentage of wild-type (driver control) follicle cells that incorporated EdU decreased as egg chambers developed from stages 4 to 6 and was maintained at a low level through stage 7 as endocycling begins (Figure 4, A, C, and E). In contrast, knock-down of *ftz-f1* was sufficient to significantly deregulate EdU incorporation in multiple developmental stages (Figure 4, B, D, and E). Moreover, the percentage of EdU-positive follicle cells in *ftz-f1^KK^* and *ftz-f1^HMS^* RNAi egg chambers remained higher than stage-matched wild-type cells through stage 7 (Figure 4E). Furthermore, we detected a small percentage of *ftz-f1^RNAi^* follicle cells in which the timing of mitotic divisions (characterized by the presence of pHH3 antisera) was extended into stages 6 and 7, suggesting that they fail to exit the mitotic cell cycle (Figure 4, D and F). Although this result only reached statistical significance for the *ftz-f1^HMS^* transgene, we also found pHH3-positive follicle cells at stage 6 in *ftz-f1^KK^* and *ftz-f1^JF^* transgenes. This contrasts with wild-type follicle cells, where pHH3 was never detected after stage 5 (Figure 4, C and F). We conclude that *ftz-f1* promotes timely exit from the mitotic cell cycle in main body follicle cells. Though this does not rule out the possible effects of other cellular processes, failure to exit the mitotic cell cycle may be a contributing factor to the premature egg chamber death in the *tjGal4>ftz-f1^RNAi^* model.

### Accumulation of oocyte-specific proteins depends on *ftz-f1* in germ cells

**Figure 5:**
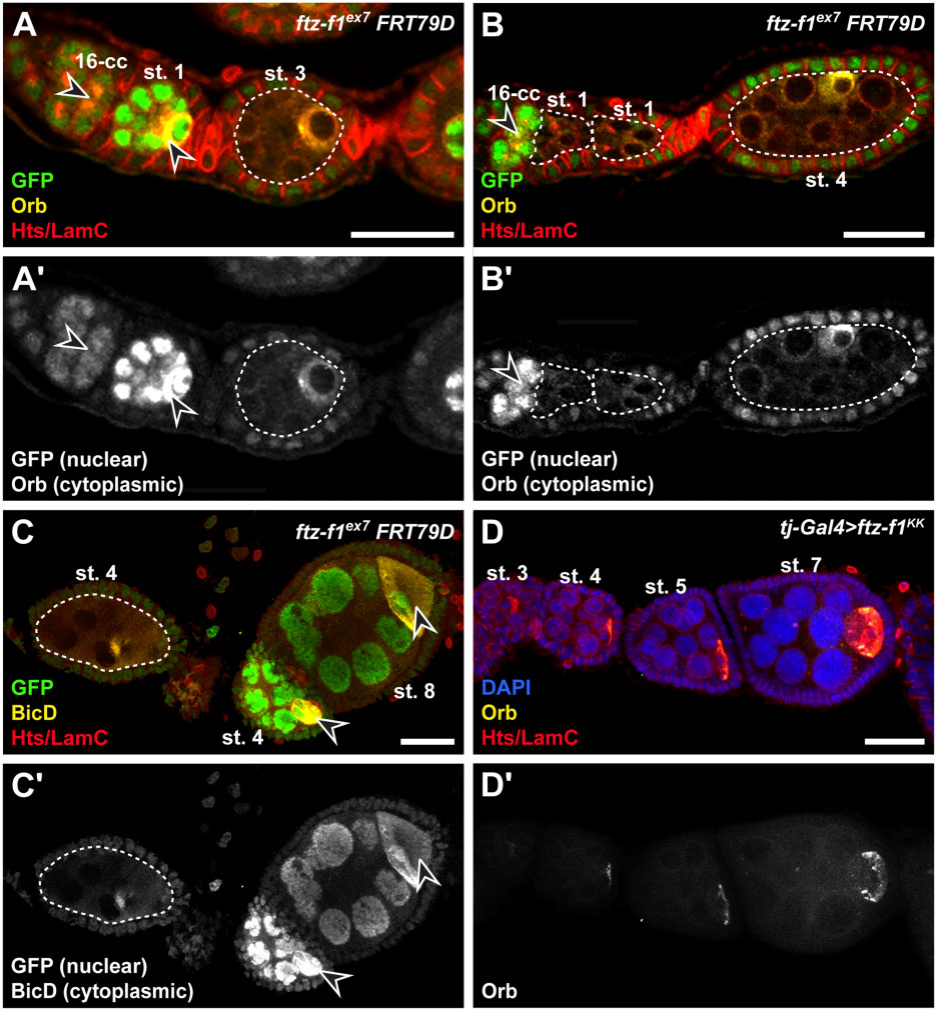
*ftz-f1* promotes accumulation of oocyte-specific factors cell autonomously in germ cells. (A–C’) Maximum intensity projections (5 μm z-plane) of representative *ftz-f1^ex7^ FRT79D* mutant mosaic germaria labeled with anti-GFP (green; wild-type cells), anti-Hts (red; fusomes and follicle cell membranes), and anti-LamC (red; nuclear envelopes). Oocytes were visualized in mosaic germaria using anti-Orb (yellow; A–B) or anti-BicD (yellow; C). *ftz-f1^ex7^* mutant cysts are outlined in dotted white lines; open arrowheads denote GFP-positive (wild-type) cysts. Panels A’–C’ depict the green channel (cytoplasmic Orb or BicD and nuclear GFP) of the images above. (D, D’) Single z-plane image of a representative ovariole from a *tj-Gal4>ftz-f1^JF^* female, immunostained for anti-Orb (yellow), anti-Hts and anti-LamC (red), and DAPI (blue). Panel D’ depicts the green channel (cytoplasmic Orb) of the image above. Scale bars, 20 μm.

Expression of Ftz-f1::GFP in GSCs and mitotically dividing germ cells suggested that *ftz-f1* could also be necessary in early germ cells for their development. In *Drosophila* germline cysts, oocyte differentiation occurs concurrently with completion of the mitotic program (Hinnant *et al.* 2020). Accumulation of oocyte-specific proteins, such as Oo18 RNA-binding protein (Orb) and Bicaudal D (BicD), in the presumptive oocyte occurs when mitotic divisions are completed and is essential for establishing oocyte polarity. Orb protein localizes near the fusome in most cystocytes in wild-type 16-cell cysts just after the last mitotic division, becoming enriched specifically in the single oocyte as pre-follicle cells migrate around the cyst to initiate egg chamber formation (arrowheads in Figure 5, A and A’). Orb levels then increase specifically in the posteriorly-positioned oocyte as the cyst grows through stages 1–5. We therefore used Orb and BicD expression as indicators of the oocyte fate and asked whether *ftz-f1* mutant cystocytes could properly differentiate. To assess egg chamber development, we again turned to the *Flp/FRT* mosaic recombination system to generate *ftz-f1* mutant germ cells in the same ovariole adjacent to wild-type egg chambers. Although GFP-negative *ftz-f1* mutant cysts (outlined in Figure 5, A–C’) expressed Orb, the levels of Orb protein in *ftz-f1* mutant cysts were much lower than in adjacent, less-developed wild-type cysts (Figure 5A; compare arrowhead in the stage 1 wild-type cyst with the outlined stage 3 mutant cyst). Most *ftz-f1* mutant cysts contained a cell with a condensed, under-replicated nucleus, consistent with typical oocyte nuclear morphology; however, oocytes were frequently mispositioned and expressed low levels of Orb (Figure 5, B and B’) and BicD (Figure 5C). These data suggested that *ftz-f1* is necessary in germ cells, independent of its role in follicle cells, for proper oocyte development. Indeed, *ftz-f1^RNAi^* in follicle cells in did not impact Orb expression (Figure 5D), suggesting that the effect of *ftz-f1* on oocyte positioning is autonomous to the germline.

## Discussion

Although *ftz-f1* is evolutionarily conserved with essential regulators of reproduction in mammals and necessary for early embryonic anterior-posterior patterning in *Drosophila*, it has been unclear whether or how *ftz-f1* impacts female reproductive function in early to mid-oogenesis. In this study, we show that *ftz-f1* is expressed throughout the ovarian epithelium and required in follicle cells for female fecundity. We show that *ftz-f1* is required in somatic cells to protect germ cells from premature caspase-dependent cell death at mid-oogenesis. We provide evidence that *ftz-f1* in follicle cells regulates the timing of mitotic exit at mid-oogenesis, coincident with down-regulation of Ftz-f1::GFP reporter expression in follicle cells at this timepoint. We conclude that Ftz-f1 normally promotes cell cycle progression in follicle cells, and that its down-regulation at stage 6 permits timely mitotic exit and egg chamber survival. Our studies also suggest that *ftz-f1* is necessary in early germ cells to promote accumulation of oocyte-specific polarity factors and suppress germ cell death. Taken together with a recent study showing that *ftz-f1* is required for ovulation (Knapp *et al.* 2020), our study adds to a growing body of literature showing that critical roles of the NR5A family are conserved from mammalian to *Drosophila* oogenesis. In particular, loss-of-function studies suggest that NR5A family members are essential in a specific spatiotemporal sequence in ovarian somatic cells for the regulation of oocyte development (Luo *et al.* 1994; 1995; Buaas *et al.* 2012; Bertolin *et al.* 2014; Meinsohn *et al.* 2018). Like *SF-1* and *LRH-1*, *ftz-f1* is necessary for proper somatic cell shape and maintenance of the somatic epithelium and may affect somatic cell proliferation and cell survival independently of its role in ovulation (Duggavathi *et al.* 2008; Buaas *et al.* 2012; Meinsohn *et al.* 2018; Knapp *et al.* 2020). Further studies will be necessary to fully elucidate the intricate molecular networks by which *ftz-f1* regulates oocyte development. Given the level of structural and functional conservation between *Drosophila* and mammalian nuclear receptor signaling pathways, we propose that *ftz-f1* will provide an excellent model for better understanding of how interactions between nuclear receptors promotes optimal female fertility.

One of the most intriguing aspects of our study is the finding that *ftz-f1* is necessary to sustain egg chamber viability through mid-oogenesis. Stages 6–8 of oocyte development are a key decision point during oogenesis (Peterson *et al.* 2015). During these stages, the oocyte begins to take up yolk, whereas follicle cells initiate a concurrent mass cell migration to cover the expanding surface area of the oocyte (McLaughlin and Bratu 2015; Duhart *et al.* 2017). Vitellogenesis is an energetically-intensive process for females; for example, ovaries from females deprived of nutrients (particularly yeast and protein) arrest at stages 6–8 of oogenesis as egg chambers undergo programmed cell death (Jenkins *et al.* 2013; Peterson *et al.* 2015; Mirth *et al.* 2019). This nutrient-mediated developmental checkpoint likely saves valuable resources, preserving fertility until the nutritional environment improves (Peterson *et al.* 2015; Sieber and Spradling 2017; Mirth *et al.* 2019). It is thus tempting to speculate that Ftz-f1 may participate in the mid-oogenesis nutritional checkpoint. At present, we cannot clearly distinguish whether the increased egg chamber death in our *tjGal4>ftz-f1^RNAi^* models is the result of caspase-independent follicle cell death (Figure 3), defective mitotic exit (Figure 5), or other cellular changes in follicle cells that disrupt soma-germline intercellular signaling. Future studies will investigate whether Ftz-f1 cooperates with other known regulators of the mid-oogenesis checkpoint, including well-known nutrient signaling pathways such as insulin signaling.

It is noteworthy that regulation of the mid-oogenesis developmental checkpoint is also controlled by ecdysone signaling, a critical regulator of insect development and fecundity. In insects, ecdysone controls gonad development and function, as well as the timing of key stages of the life cycle (Uryu *et al.* 2015; Ables and Drummond-Barbosa 2017; Swevers 2019). Like mammalian reproductive hormones, ecdysone is synthesized in developing ovarian follicles in adults (Uryu *et al.* 2015). Cellular responses to ecdysone are mediated by a heterodimeric nuclear receptor complex consisting of EcR and Ultraspiracle (Usp). Although the transcriptional response to EcR/Usp is extensive, several key targets have been identified, including the nuclear receptors encoded by *E75* and *Ecdysone-induced protein 78C* (*E78*) and the transcription factors encoded by *broad* (*br*) and *Ecdysone-induced protein 74EF* (*E74*) (Yamanaka *et al.* 2013; Stoiber *et al.* 2016; Uyehara and McKay 2019). EcR/Usp controls the nutritional checkpoint for progression past stage 8 and is essential for vitellogenesis and eggshell formation, making this complex critical for follicle survival (Buszczak *et al.* 1999; Carney and Bender 2000; Sieber and Spradling 2015). Moreover, *E74*, *E75*, and *E78* are necessary for follicle survival and are thought to cooperate with EcR/Usp (Buszczak *et al.* 1999; Terashima and Bownes 2006; Ables and Drummond-Barbosa 2010; Ables *et al.* 2015). Nuclear receptor function apparently converges at mid-oogenesis, suggesting that a complex network of molecular interactions between Ftz-f1, EcR, E78, and E75 may collectively promote egg chamber survival.

Additional studies will be necessary to tease apart whether and how Ftz-f1 participates in the ecdysone signaling network at mid-oogenesis. Some clues to uncovering this interaction can potentially be gleaned from studies of Ftz-f1 in larval cells as they prepare for metamorphosis. In pre-pupae, *ftz-f1* transcription is repressed by ecdysone (Woodard *et al.* 1994; Rewitz *et al.* 2010). Yet expression of *ftz-f1* in the presence of ecdysone enhances transcription of *E74*, *E75*, and *br* (Woodard *et al.* 1994; Broadus *et al.* 1999; Zhu et al. 2006; Ruaud *et al.* 2010). Previous studies have thus suggested that Ftz-f1 functions as a competence factor, setting up a transcriptional program when ecdysone levels are low that permits activation of ecdysone signaling at later points of development (Broadus *et al.* 1999). One possibility is that Ftz-f1 is indirectly influenced by an ecdysone-induced transcription factor, such as EcR, E74, E75, and/or E78, to promote mid-oogenesis survival. Unfortunately, it has been difficult to tease apart the interactions between these factors, given the high degree of egg chamber death in loss-of-function mutants. Our recent identification of cis-regulatory enhancers located in ecdysone response genes (McDonald *et al.* 2019) may prove to be useful reagents to analyze these complex genetic interactions.

Another possibility is that Ftz-f1 may promote egg chamber survival by activating the transcription of steroid hormone biosynthesis genes. Previous studies showed that *ftz-f1* is required in the larval ring gland for ecdysone synthesis via cytochrome P-450 enzymes (Parvy *et al.* 2005; 2014; Borsos *et al.* 2015). As applied to the ovary, this is an intriguing hypothesis worthy of additional study. The ovary produces ecdysone in response to maternal nutrition and ecdysone biosynthesis genes are necessary for egg chamber survival at mid-oogenesis (Buszczak *et al.* 1999; Warren *et al.* 2002; Petryk *et al.* 2003; Ono *et al.* 2006; Uryu *et al.* 2015). But in contrast to the well-studied prothoracic gland (Ou and King-Jones 2013; Ou *et al.* 2016; Uryu *et al.* 2018), transcriptional regulation of the biosynthesis enzymes in the ovary remains largely unknown (Uryu *et al.* 2015). Mammalian NR5A receptors LRH-1 and SF-1 are essential for steroid hormone biosynthesis, suggesting that Ftz-f1 may likewise promote ecdysone biosynthesis (Meinsohn *et al.* 2019). Future studies investigating how ecdysone biosynthesis is regulated by nuclear receptors in the *Drosophila* ovary will provide an intriguing genetically tractable model to study how maternally derived nutrients and maternal physiology promote female fertility, with potential applications for fertility preservation in humans.
